# Effects of Different Substrates on the Formability and Densification Behaviors of Cemented Carbide Processed by Laser Powder Bed Fusion

**DOI:** 10.3390/ma14175027

**Published:** 2021-09-02

**Authors:** Decheng Liu, Wen Yue, Jiajie Kang, Chengbiao Wang

**Affiliations:** 1 School of Engineering and Technology, China University of Geosciences (Beijing), Beijing 100084, China; ldccugb@sina.com (D.L.); cbwang@cugb.edu.cn (C.W.); 2 Beijing Geology and Mineral Resources Prospecting and Developing Company, Beijing 100050, China; 3 Zhengzhou Research Institute, China University of Geosciences (Beijing), Zhengzhou 451283, China

**Keywords:** laser powder bed fusion (L-PBF), cemented carbide, Ni200 substrate, densification, crack

## Abstract

Cemented carbide materials are widely applied in cutting tools, drill tools, and mold fabrication due to their superior hardness and wear resistance. Producing cemented carbide parts via the laser powder bed fusion (L-PBF) method has the advantage of fabricating complex structures with a rapid manufacturing speed; however, they were underdeveloped due to their low density and crack formation on the blocks. This work studied the effect of different substrates including 316L substrates, Ni200 substrates, and YG15 substrates on the forming quality of WC-17Co parts fabricated by L-PBF, with the aim of finding the optimal substrate for fabrication. The results revealed that the Ni200 substrates had a better wettability for the single tracks formation than other substrates, and bonding between the built block and the Ni200 substrate was firm without separation during processing with a large range of laser energy inputs. This guaranteed the fabrication of a relatively dense block with fewer cracks. Although the high laser energy input that led to fine crack formation on the blocks formed on the Ni200 substrate, it was found to be better suited to restricting cracks than other substrates.

## 1. Introduction

Due to their superior hardness, anti-thermal expansion, and excellent wear-resistance, cemented carbide parts were widely applied in cutting tools, geographical engineering, and mold fabrication [1,2,3]. Generally, the cemented carbide part was produced by the powder metallurgy (PM) method. In this method, the cemented carbide powders (i.e., the mixture powder of WC powders and binder powders such as Co, Ni, Fe) were put into designed molds and sintered under high-temperature and high-pressure conditions to fabricate the part [4,5]. Constrained by the high-pressure processing conditions in the traditional PM method, the mold could not be too complex in shape. In addition, the finished product was difficult to machine due to its superior hardness. Thus, there are significant limitations in fabricating cemented carbide with complex shapes.

Laser powder bed fusion (L-PBF), also known as selective laser melting (SLM), is one of the most important branches of metal additive manufacturing (AM) techniques, providing an ideal platform for the design and production of complex structural parts with a near net shape [6]. Compared with the traditional method of casting and forging, L-PBF utilizes the powder bed system to supply the metallic powder and laser beam to selectively melt metallic powder layer by layer according to the 2D contour from a 3D CAD model; so, it could theoretically build metal parts with any complicated structures, unlike conventional processing technology [7]. Thus far, L-PBF technologies have been successfully applied to manufacture dozens of kinds of metal materials, including stainless steels [8] and Al-based [9,10], Ti-based [11,12], and Ni-based materials [13,14].

The great advantages of L-PBF during the processing of complex structural parts has led to the formability and printability of cemented carbide parts by L-PBF receiving extensive attention. Some studies have reported on the L-PBF fabrication of cemented carbide parts [15,16,17,18,19]. Uhlmann et al. [15] investigated the forming quality of the L-PBF WC-Co part with different Co content within a certain range of laser energy density. The result discovered that cracks were observed under the entire range of laser energy density, although high laser energy density would improve the relative density for all kinds of parts. In addition, Co evaporation was induced during the fabricating process at the same time. This phenomenon decreased the toughness of the part and promoted the fine cracks to grow into more continuous cracks. Liu et al. [16] found that the WC grains had an uneven growth due to the uneven distribution of sintering temperature, high cooling rate, and remelting phenomenon during L-PBF processing. Gu et al. [17] studied the formation mechanism for L-PBF WC/Ni2W4C(M6C) cemented-carbide-based materials and obtained a cemented carbide block with a relative density of 96.3%. In this study, cracks and pores were found on the specimens. Li et al. [19] selected the 20 wt % NiAlCoCrCuFe high-entropy alloy powder as the binder when fabricating cemented carbide by L-PBF technology, which proved to be conducive to obtaining high dense blocks, but they did not solve the serious delamination phenomena for the samples. These studies demonstrated that L-PBF could process the cemented carbide parts, but some defects have not been avoided yet. Pores, delamination, and cracks formed during processing, which are caused by the remarkable embrittlement of the cemented carbide and high thermal stress during laser processing [19,20]. These defects reduce the densification and weaken the integrity of the part, particularly for cracks and delamination, which significantly affect the mechanical properties and limit the L-PBF cemented carbide part from being applied to actual engineering. Therefore, the restriction of the pores and cracks during L-PBF processing of cemented carbide is critical.

As previous studies proved, laser energy input and binder properties could significantly influence the forming quality of cemented carbide parts fabricated by L-PBF. In addition, the substrates, as the initiation of the materials’ deposition, also played an important role in the L-PBF manufacturing process [21,22,23]. The wettability between the powders and substrates during the processing could determine the forming quality of final parts. Liu et al. [23] found that the flowability and wettability of liquid materials in the molten pools were significantly affected by the substrate surface morphologies, thereby affecting the final forming quality of the L-PBF part. Khmyrov et al. [24] adopted the SiO_2_ materials as a substrate when fabricating SiO_2_ single tracks by L-PBF. Their results uncovered that this substrate was beneficial for lowering residual stress and well-wetting the powders during the fabricating process. Thus, regular single tacks with almost no cracks were formed on the substrate. The effect of substrate on the forming quality of L-PBF cemented carbide parts has not been reported. Finding a suitable substrate may improve the forming quality of the L-PBF cemented carbide part.

In this study, three different substrates were used in the L-PBF processing of cemented carbide. To uncover the effect of the substrates on the forming quality of the specimens, single tracks and blocks were fabricated on these substrates under different process parameters; then, the morphology, geometrical characteristics, and densification behaviors of these samples were analyzed to find the most suitable substrate for fabricating the cemented carbide part.

## 2. Materials and Methods

### 2.1. Powder Materials

**Figure 1:**
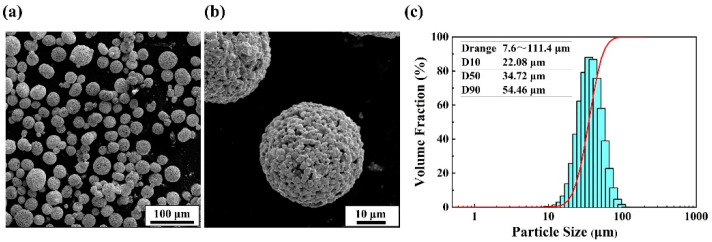
WC-17Co powders: (**a**) the low-magnification SEM micrographs of powders, (**b**) the high-magnification SEM micrographs of powders, and (**c**) the particle size distribution of powders.

The cemented carbide powders used in this study were WC-17Co powders (Shenyang Institute of Nonferrous Metals, Shenyang, China) fabricated by the agglomeration and sintering method, which can achieve high-quality metallurgical bonding between Co powder and WC powder. As shown in Figure 1, the powders present a near-spherical shape with typical metallurgical characteristics of cemented carbide powder, and the particle size distribution of powders were D10 (22.08 μm), D50 (34.72 μm), and D90 (54.46 μm). The chemical composition of the powders is shown in Table 1.

### 2.2. Substrates

According to the similarity and the wettability of the WC-17Co materials as well as the production cost, three kinds of homemade substrates made of 316L stainless steel material (316L substrate—contains the dominant Fe element, which has good wettability to cemented carbide), Ni200 pure nickel material (Ni200 substrate—contains the dominant Ni element, which has good wettability to cemented carbide), and YG15 cemented carbide material (YG15 substrate—has the similarity element component to WC-15Co) were used in L-PBF processing of WC-17Co powders. These substrates were grinded by a grinding machine to make the surface flat and smooth with the surface roughness (Ra) of 4.45–5.46 μm and were fixed horizontally at the forming platform before L-PBF processing.

### 2.3. L-PBF Processing

**Figure 2:**
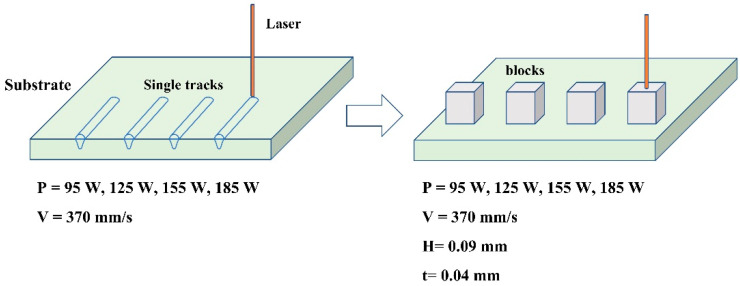
Schematic diagram of the WC-17Co samples prepared by L-PBF.

An L-PBF system, the EOS M280 (Germany) with a 400-W fiber laser, was used to prepare single tracks and blocks on the different substrates. The processing parameters are listed in Figure 2. The single tracks with a length of 10 mm were fabricated with the following processing parameters: a laser beam scanning speed (V) of 370 mm/s, layer thickness (t) of 40 μm, and the power of laser beam (laser power, P) from 95 to 155 W with an interval of 30 W. The corresponding blocks were printed at the constant hatching distance (H) of 90 μm; the dimension of the blocks was 8 × 8 × 4 mm^3^.

### 2.4. Characterization

To understand the joint mechanism between samples and different substrates, the single tracks were cut by wire cutting along the building direction to obtain the cross-section of single tracks. Each specimen was first mechanically ground with SiC paper to 2000 grit and then polished up with a mirror finish to observe the formation and geometrical characteristics of molten pools. Scanning electron microscope (SEM) (Phenom-World, Eindhoven, The Netherlands) was used to characterize the surface morphologies of single tacks and blocks. The density of the blocks was measured by the Archimedes method.

## 3. Results

### 3.1. Surface Morphologies of Single Tracks

**Figure 3:**
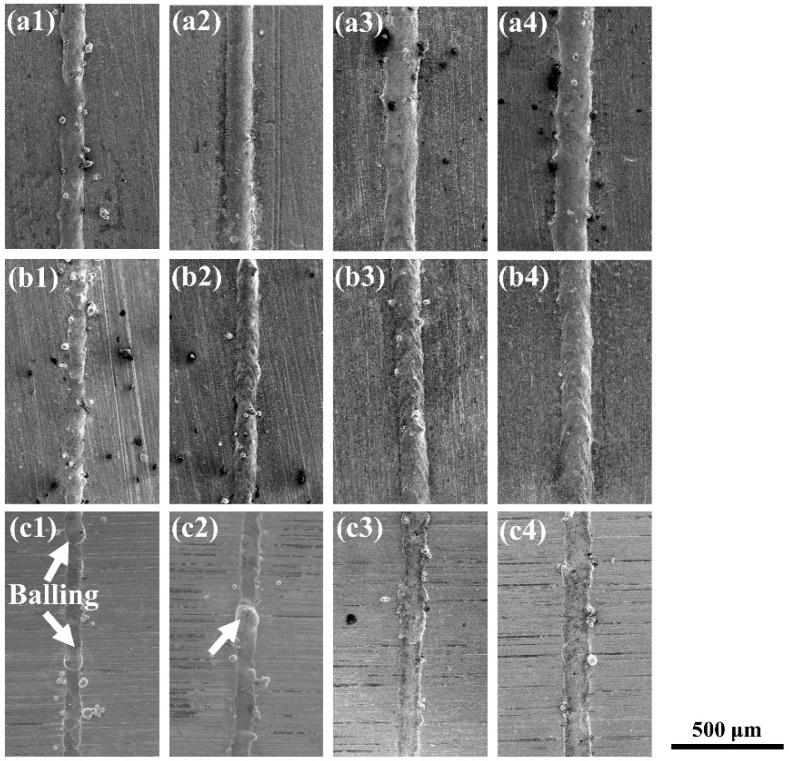
Single tracks formed on different substrates: (**a1**–**a4**) single tracks formed on the 316L substrate with laser power from 95 to 185 W; (**b1**–**b4**) single tracks formed on the Ni200 substrates with laser power from 95 to 185 W; (**c1**–**c4**) single tracks formed on the YG15 substrate with laser power from 95 to 185 W.

**Figure 4:**
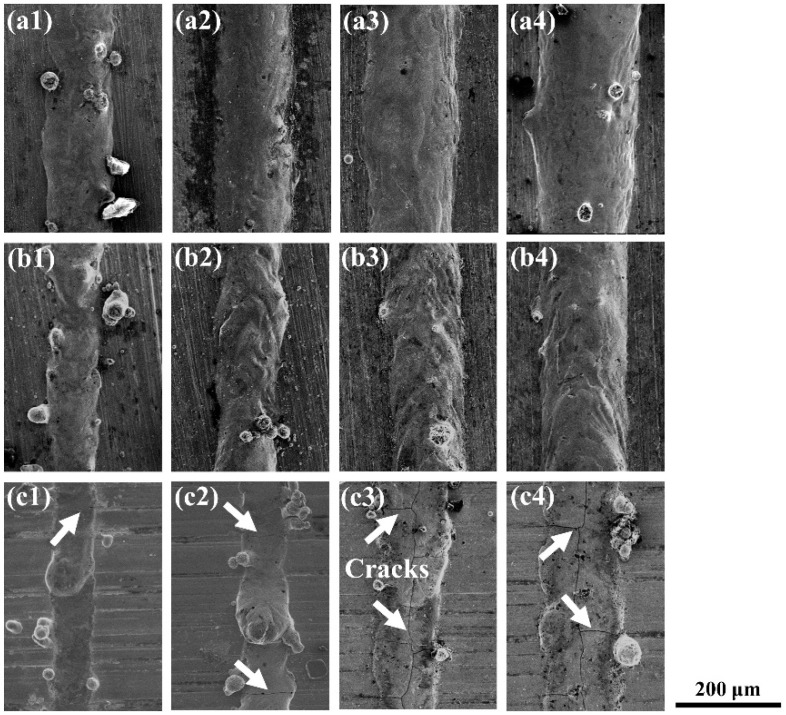
The high-magnification SEM micrographs of single tracks corresponding to Figure 3, (**a1**–**a4**) single tracks formed on the 316L substrate with laser power from 95 to 185 W; (**b1**–**b4**) single tracks formed on the Ni200 substrates with laser power from 95 to 185 W; (**c1**–**c4**) single tracks formed on the YG15 substrate with laser power from 95 to 185 W; the red arrows indicate the cracks on single tracks.

The single tracks formed under different laser powers are exhibited in Figure 3. It was found that the morphology of the single tracks varied with the laser powers and the substrates. The single tracks formed on the YG15 substrate presented a flat surface morphology. According to previous studies, the laser-melted metal materials during L-PBF processing were similar to those of the other laser processing method, which generate liquid molten pools on substrates and form approximately semicylindrical single tracks along the scanning direction of laser scanning under the surface tension [25]. The single tracks formed on the YG15 substrate look like thin slices that stick to the substrate in all processing parameters. This phenomenon proved that there was insufficient metal liquid in the molten pool. The balling effect (indicated by white arrows in Figure 3) appeared at the laser powers of 95 and 125 W and disappeared at 155 and 185 W. In addition, some cracks were produced on the single tracks. Figure 4 shows the high-magnification SEM micrographs of single tracks and reveals that all single tracks formed on the YG15 substrate suffered cracks (indicated by white arrows in Figure 4), and the quantities and sizes of cracks increased when the laser power increased. In comparison, no cracks were observed on the single tracks formed on other substrates. These phenomena illustrate that the YG15 substrate might be not a suitable selection for the fabrication of the WC-17Co part.

It can be seen that the single tracks formed on the Ni200 substrate and 316L substrate have many similarities, but all of them were distinctly different to those formed on the YG15 substrate. The single tracks formed on the Ni200 substrate and 316L substrate presented the typical surface morphology of single tracks, as similarly shown in previous studies [25]. These single tracks were regular and continuous. Even at low laser power, there were only some unmelted powders around the single tracks, and balling behaviors were not observed. When the laser power was high, the unmelted powders significantly decreased, and the morphology of the single tracks became smooth and uniform. Furthermore, cracks did not form on these single tracks. The above results exhibit that single tracks with excellent surface morphology can be fabricated on 316L and Ni200 substrates, indicating that they have the potential to be used to fabricate WC-17Co blocks successfully.

**Figure 5:**
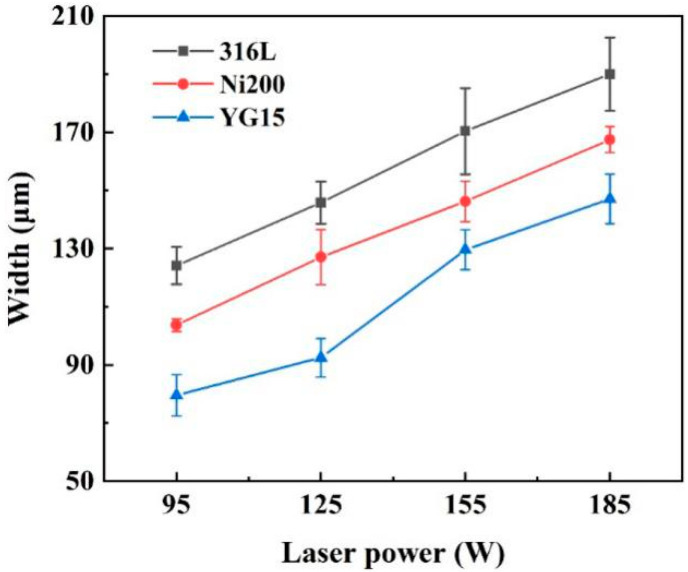
Widths of the single tracks formed at different laser powers.

Figure 5 shows the width of the tracks formed on different substrates. It was found that the width of single tracks also varied with the laser power and substrates. At the same laser power, the single tacks formed on the YG15 substrate present the smallest width. At 95 W, the width was even below 80 μm, which was much lower than the laser spot size (around 100 μm). With the increase in laser power, the width of single tracks increased but was lower than that of other substrates. These narrow single tracks led to an insufficient overlap ratio between adjacent laser tracks during block preparation. In contrast, the single tracks on 316L substrates and Ni200 substrates had larger widths, which was beneficial to the following fabrication of blocks. Meanwhile, the single tracks on 316 substrates were wider than those on Ni200 substrates when the laser power was consistent. This difference illustrates that the deposition of WC-17Co materials on the two substrates would also have some differences.

### 3.2. Forming Characteristics of Molten Pools

**Figure 6:**
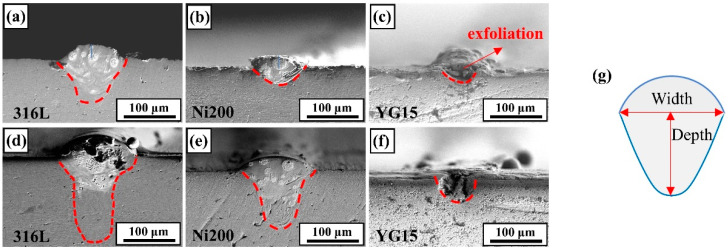
The cross-section of the single tracks: (**a**–**c**) the single tracks formed at 125 W; (**d**–**f**) the single tracks formed at 185 W; (**g**) the schematic diagram of the molten pool size.

**Figure 7:**
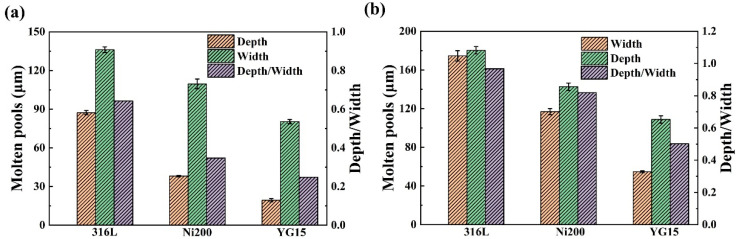
The molten pool size: (**a**) the size of the molten pool formed at a laser power of 125 W; (**b**) the size of the molten pool formed at a laser power of 185 W.

The forming and geometric characteristics of molten pools were observed through polishing the cross-sections of the single track on the substrates, as shown in Figure 6. It also shows that the forming and geometrical characteristics were distinctly different for these molten pools. On the YG15 substrates, the molten pools presented an inferior forming quality, which mainly reflects poor bonding with the substrate. During the polished processing, the molten pools were easily peeled off from the cross-section, as shown in Figure 6c, meaning the bonding between the substrate and molten pools was weak. Additionally, Figure 6f exhibits some holes and cracks in the molten pools, causing the poor bonding. On the other hand, the small width and depth of the molten pool would induce insufficient remelt for the adjacent molten pools and pre-deposited layers. As a comparison, the molten pools formed on Ni200 and 316L substrates were normal, regular, and tightly bonded with the substrates. However, some differences still exist between the two kinds of molten pools. According to the results of Figure 7, the depth and width of the molten pools formed on Ni200 substrates are smaller than those of 316L substrates at the same laser power. Moreover, the disparity at the depth values is more distinct than that of width values. This phenomenon led to a different penetration depth-to-width ratio (D/W) of the molten pools, which could determine the heat conduction model of the molten pools [26]. The D/Ws of the molten pools on the 316L substrate were 0.68 and 0.98, whereas the D/Ws of the molten pools on the Ni200 substrate were 0.37 and 0.82. These demonstrate that the molten pools on 316L substrates are more likely to form the keyhole model, as shown in Figure 6d. This keyhole model molten pool was not recommended during L-PBF processing because defects such as circular pores would be easily induced by this model [26]. This result demonstrates that the Ni200 substrates could fit a more extensive range of laser energy density during the fabrication of WC-17Co parts by L-PBF.

**Figure 8:**
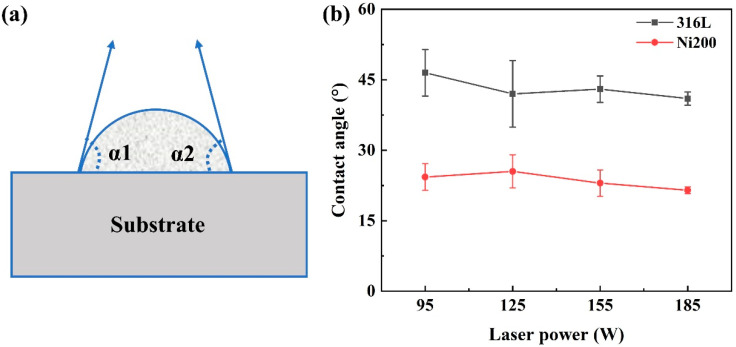
(**a**) Schematic diagram of the contact angle. (**b**) Contact angle of the single tracks formed on 316L and Ni200 substrates.

The contact angle of the single tracks reflects the wetting ability between the liquid metals in molten pools and the solid substrates [23]. The lower the value of the contact angle, the better the wettability. Considering the good forming quality of the single tracks on Ni200 and 316L substrates, the contact angle (α, °) of these single tracks is obtained from Formula (1) [27], and the variation tendency is shown in Figure 8.

(1) $$\alpha =\frac{\left(\alpha 1+\alpha 2\right)}{2}$$

where α1 and α2 represent the contact angle of the left and right region. It was found that the contact angle of single tracks on Ni200 substrates and 316L substrate were below 25° and 47°. According to the result of contact angle with good wettability (around or below 40°) in Liu et al. [27], both the Ni200 and 316L substrates had good wettability of WC-17Co powders in L-PBF processing. The contact angle on the Ni200 substrates was smaller when the laser power was constant, meaning that the Ni200 substrates could better wet the cemented carbide tracks than the 316L substrates. With the increase in laser power, the contact angle presented a decreasing trend in both the Ni200 and 316L substrates, meaning the high energy input also improved the wettability.

### 3.3. Blocks Preparation

**Figure 9:**
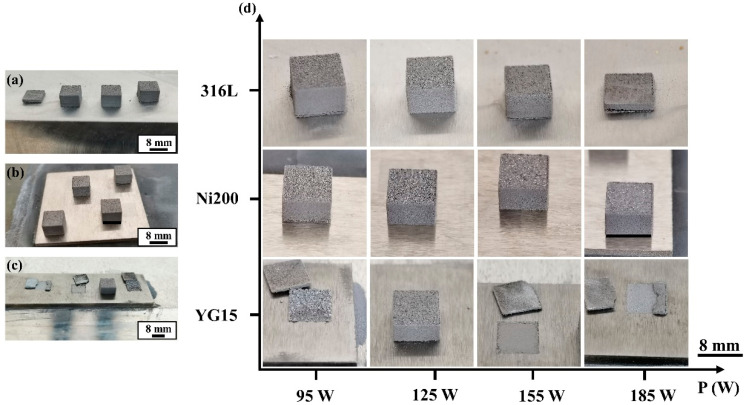
The blocks formed on different substrates: (**a**) 316L substrate; (**b**) Ni200 substrate; (**c**) YG15 substrate; (**d**) high-magnification picture of blocks.

The result of the single tracks formed on substrates revealed that there were distinct different wettability and bonding strengths between the WC-17Co materials and substrates, which directly influenced the following block fabrication. Figure 9 shows the WC-17Co blocks fabricated on different substrates, revealing that the fabrication result of the blocks is dependent on the three kinds of substrates. Compared with 316L (Figure 9a) and Ni200 substrates (Figure 9b), the YG15 substrate (Figure 9c) exhibited an extremely low success rate for the fabrication of the blocks. At 95 W, 155 W, and 185 W, the specimens on YG15 substrates failed to be fabricated at the initial stage. This was due to the serious warping that appeared at the initial stage of the forming process, hindering the movement of the blade and causing the ineffective recoating of powder. Once these defects appeared, the processing status would continuously worsen and induce more excessive warp, making the blade peel off the specimens from the substrates. Although the remaining specimen (formed at 125 W) was integrally fabricated, obvious cracks were observed between the sample and the substrate in the high-magnification pictures (Figure 9d), proving that this fabrication was also a failure. When the blocks were fabricated on the 316L substrate, more quantities of integrated WC-17Co blocks were obtained, meaning the forming quality of these samples was better than those formed on the YG15 substrate. However, long and durative cracks inevitably formed between the substrate and the samples at 95 and 155 W, and the premature failure of sample fabricating was found at 185 W. These phenomena illustrated that the 316L substrate was also unsuitable for L-PBF processing of WC-17Co blocks. When Ni200 materials were used as the substrate, none of the fabricated WC-17Co blocks suffered a serious warp during processing, making all samples successfully fabricated. From the high-magnification pictures of blocks, it was found that these blocks formed in the range of 125–185 W were tightly bonded with the Ni200 substrate without any well-marked cracks between the sample and the substrate. The cracks that appeared at the junction of the sample and the substrate with a laser power of 95 W, similar to the sample fabricated on the 316L substrate at the same laser power, were attributed to the improper processing parameter. In contrast with previous studies [19,20,28], no delamination appeared on the blocks formed on the Ni200 substrates, meaning the selection of process parameters and Ni200 substrates has the potential to form high-quality WC-17Co parts.

### 3.4. Surface Morphologies and Densification Behaviors

**Figure 10:**
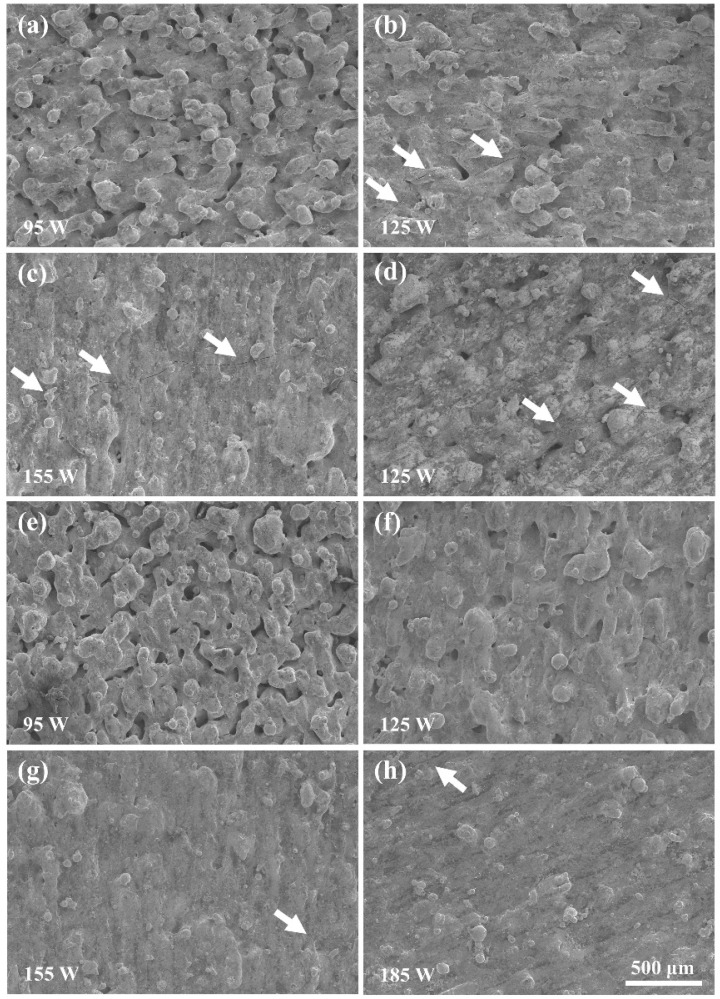
The SEM morphologies of the bulk surface: (**a**–**c**) bulks formed on the 316L substrates; (**d**) bulk formed on the YG15 substrate; (**e**–**h**) bulks formed on the Ni200 substrate.

According to the result in Figure 10, the top surface morphology of the prepared blocks was characterized by SEM to uncover the variation of the blocks’ forming quality. Figure 10a–c exhibit the blocks fabricated on the 316L substrate at 95 W, 125 W, and 155 W. At 95 W, the blocks presented extremely uneven and irregular surface morphology. The serious balling effect and humps disrupted and distorted the laser tracks, thus producing an almost ineffective overlap of the adjacent laser tracks and leading to larger quantities of pores. When the laser power was increased, the balling effect and hump were effectively restrained, and laser tracks were regular and continuous. Thus, the surface became smooth and the quantities of the pores sharply decreased. At 155 W, the pores almost disappeared, meaning dense blocks would be achieved at a high laser energy input. However, the cracks (indicated by white arrows) gradually became more serious with the increase in laser power. At 155 W, long and obvious cracks appeared on the surface.

**Figure 11:**
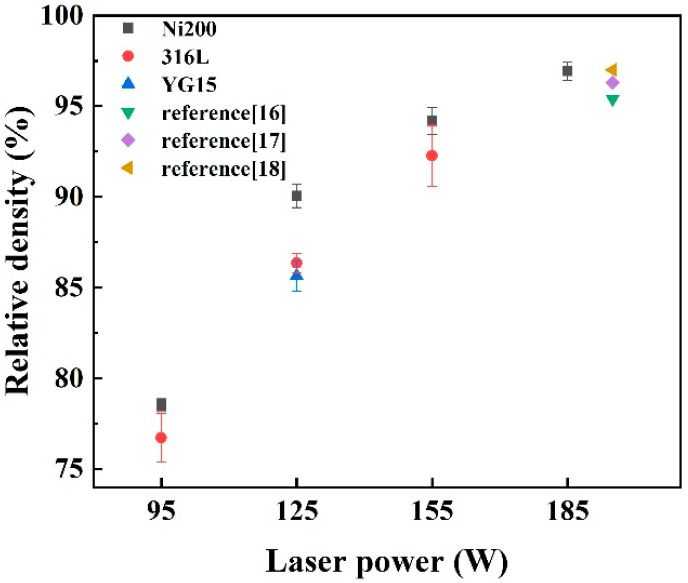
Relative densities of the integrated blocks.

The only integrated block was prepared on the YG15 substrate at a laser power of 125 W. The surface was coarse and had obvious pores and cracks due to the low laser energy input. According to Figure 10e–h, the blocks on the Ni200 substrate displayed a similar surface morphology to the blocks on 316L substrates, which varied with laser powers. The higher laser power led to a smoother surface with fewer pores. In addition, the cracks on the blocks were not as serious as those shown on the block that formed on 316L substrates at the same laser power. However, the increase in laser power also led to more cracks on the surface; this was consistent with the result of Uhlmann et al. [15]. Figure 11 shows the relative densities of these blocks. The result that blocks formed on different substrates at the same power had an approximate relative density was found; the relative density of blocks on the Ni200 substrate was better. The relative densities improved with the increase in laser energy input. At the laser power of 185 W, the block achieved an optimal relative density of 96.94%, which was superior to or close to the result (around 95.40–96.97%) in the previous studies [16,17,18] about the similar WC-Co part that was fabricated by L-PBF.

## 4. Discussion

### 4.1. The Effect of Substrates on Molten Pool Formation

**Figure 12:**
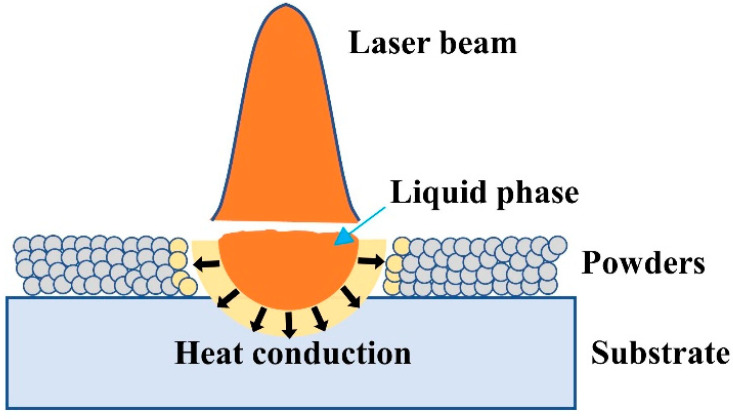
Schematic diagram of heat conduction during molten pool formation.

According to the above experimental result of single tracks, the three kinds of substrates exhibited different wettability and bonding strengths with the WC-17Co materials. This was attributed to the different physical properties of the substrates—including the melting point and thermal conductivity—that induced distinct differences in the thermal behaviors during the fabrication. As shown in Figure 12, with the laser irradiated on the WC-17Co powders, huge energy was absorbed by the powders, and the temperature of the powders rapidly increased to exceed its melting point, thus forming a liquid phase. At the same time, the heat quickly transferred to the substrate to form a molten pool. When the samples were fabricated on the YG15 substrate, the substrate presented a much higher melting point (approximately 2273.15 K) than that of the 316L (approximately 1673.15 K) and Ni200 substrates (approximately 1723.15 K). This would make a small quantity of the substrate metal convert into the liquid phase. The liquid phase in the substrate, commonly called the lower melt pool, plays an important role in suppressing the balling caused by the upper melt pool (the liquid phase in the powders layer). When the lower melt pool had insufficient liquid materials, such as the YG15 substrate here, it would not effectively hinder the balling trend of the upper part, thus promoting balling effects and lowering the wettability of the liquid phase to the substrate [29,30]. The YG15 substrate has excellent heat conduction properties (thermal conductivity around 82 W/m·K); this produces a higher cooling rate of the molten pool, which was prone to the cracks’ initiation [31]. Moreover, the insufficient amount of liquid could not be ensured to backfill the cracks efficiently; thus, the solidification characteristics of the single tracks were inferior [29]. The lower melting point of the Ni200 and 316L materials resulted in much more liquid material in the molten pools that formed on the Ni200 and the 316L substrates. Not only did the sufficient liquid phase have good flowability and wettability for the substrates to decrease the balling effect, it also prolonged the solidification time of the molten pool, thereby forming wide single tracks with better surface morphology [32]. In addition, the Ni200 (thermal conductivity around 67 W/m·K) has a better heat conduction property than 316L stainless (thermal conductivity around 15 W/m·K). This would make the heat of the molten pool easily conduct more materials, leading to greater heat loss. The size of the molten pool would be smaller, thus preventing formation of the keyhole model pool at high laser energy input.

As in previous studies, Fe, Ni, and Co belong to one family element, meaning a good wettability to the WC phase; thereby, Fe and Ni were also used as the binders of cemented carbide [33,34]. The contact angle of the single tracks formed on 316L substrates and Ni200 substrates was low. However, compared with Ni, Fe has a worse wettability to the WC phase and more easily forms a brittle phase with WC, leading to an infirm bonding between the built part and substrate, which promoted the formation of cracks during L-PBF processing of WC-17Co [34].

### 4.2. The Effect of Laser Energy Input on the Molten Pool Formation

According to the result of the molten pools formed on the Ni200 substrates, the surface morphology and geometrical characteristics of the molten pools depend on the laser energy input. High laser energy input induces high operative temperature in the molten pools, which would melt more materials into liquid and provide a long lifetime of the molten pool, thus leading to a wide molten pool [35]. In addition, the dynamic viscosity ($\eta_{v}$) (Pa·s) of the molten pool was mainly determined by the operative temperature in the molten pool, as expressed in Formula (2):

(2) $$\eta_{v}=\frac{16}{15}\sqrt{\frac{m}{kT}}\sigma$$

where m is the atomic mass (Kg); k is the Boltzmann constant (1.38 × 10^−23^ J/K); T is the molten pool temperature (K); and σ is the surface tension (N/m), which is inversely proportional to T. According to this formula, the higher the temperature, the lower the dynamic viscosity of the liquid metal and the smaller the surface tension. Therefore, good flowability and spreadability of the liquid materials were obtained when the laser energy density was high, which were beneficial to form wide molten pools with low contact angles.

### 4.3. The Effect of Laser Energy Input on Block Formation

**Figure 13:**
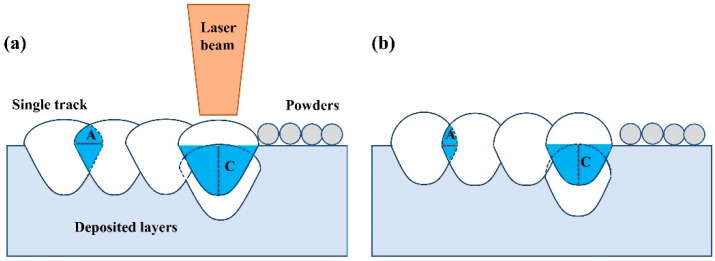
The schematic diagram of the overlap between single tracks: (**a**) the single track with large depth and width; (**b**) the single track with small depth and width.

The laser energy input induced different forming characteristics of the single tracks, thereby influencing the forming quality of the blocks. With the increase in laser energy density, the surface morphologies of the single tracks become regular and uniform. This phenomenon could help form ordered bonding track by track to improve the surface quality of the blocks formed on the Ni200 substrate. Figure 13 shows the schematic diagram of laser track bonding during block fabrication. It demonstrates that the width and depth of the single tracks directly determine the overlap portion (the blue region in Figure 13) of the adjacent tracks in the horizontal direction (indicated by A in Figure 13) and the neighboring layers in the vertical direction (indicated by C in Figure 13). The large width and depth of the molten pools formed under high laser energy input induced a sufficient overlap portion, indicating a good metallurgy bonding between single tracks [36]. Moreover, during L-PBF processing, the repeated laser scanning on the powders layer by layer not only melts the current metal powders but also remelts the deposited single tracks and layers under the continuous heat cycles; therefore, the single tracks undergo melting and solidification several times. When the adjacent tracks or layers had a large overlap portion, it led to more melting and solidification cycles for the single tracks, meaning the lifetime of the liquid molten pools was lengthened [32]. Thus, the liquid metal was spread more sufficiently to infiltrate powders and fill the holes, improving the density of the blocks.

Although the cracks on the blocks that formed on Ni200 substrates were significantly fewer than those formed on other substrates, some fine cracks still formed when the laser energy input was high. The formation of the cracks was close to the residual stress formed during rapid fabrication. When the energy input was higher, it would result in a higher cooling rate and temperature gradient, thus producing higher thermal stress, which induced further crack formation [37].

## 5. Conclusions

This study aims to find a substrate that has a good wettability to the cemented carbide during L-PBF processing. Therefore, the formability and densification behaviors of selective laser melted WC-17Co materials on different substrates were studied by fabricating the single tracks and corresponding blocks under a range of laser energy densities. The general conclusions can be summarized as follows:

(1) The high melting point of the YG15 substrate led to an insufficient liquid phase in the melt pool, thus causing an inferior forming quality of the single tracks at all process parameters; this also resulted in a poor success rate for the integrated fabrication of blocks.
(2) The low melting point of the 316L substrate and Ni200 substrate produced sufficient liquid phase, which was beneficial to forming regular and continuous single tracks. Ni200 substrates present a better wettability to WC-17Co materials than those of 316L substrates, which produce an infirm bonding between the built block and Ni200 substrate to restrict the separation of blocks and substrate at a high laser energy input.
(3) The blocks formed on the Ni200 substrates presented a better forming quality than those formed on other substrates, and a block with a relative density of 96.94% and fewer cracks was obtained at the laser power of 185 W and scanning speed of 370 mm/s.

In summary, the Ni200 substrates presented excellent adhesive bonding to the WC-17Co materials for a wide range of laser energy inputs. Furthermore, no obvious delamination and serious cracks were observed on the blocks. This supplies a more operable space to continue to optimize the process window, such as greater increases of laser energy input, the remelting strategy, and so on.

## Figures and Tables

**Table 1:** Chemical composition of the WC-17Co powders.

Elements	W	Co	C	O	Fe
wt %	bal.	17.10	5.28	0.013	0.009

## Data Availability

The raw data required to reproduce these findings cannot be shared at this time as the data also forms part of an ongoing study.
